# Mast cells modulate the inflammatory process in endotoxin-induced uveitis

**Published:** 2011-05-08

**Authors:** Pierre Sebastião da Silva, Ana Paula Girol, Sonia M. Oliani

**Affiliations:** 1 From the Post-Graduation in Morphology, Federal University of São Paulo (UNIFESP), São Paulo, SP, Brazil; 2 Department of Biology, Instituto de Biociências, Letras e Ciências Exatas (IBILCE), São Paulo State University (UNESP), São José do Rio Preto, SP, Brazil

## Abstract

**Purpose:**

To investigate the role of mast cells and annexin-A1 (Anxa1) in endotoxin-induced uveitis (EIU).

**Methods:**

EIU was induced by injection of lipopolysaccharide (LPS) into the paws of rats, which were then sacrificed after 24 and 48 h. To assess EIU in the absence of mast cells, groups of animals were pretreated with compound 48/80 (c48/80) and sacrificed after 24 h after no treatment or EIU induction. The eyes were used for histological studies and the aqueous humor (AqH) pool was used for the analysis of transmigrated cells and Anxa1 levels. In inflammatory cells, Anxa1 expression was monitored by immunohistochemistry.

**Results:**

After 24 h, rats with EIU exhibited degranulated mast cells, associated with elevated numbers of infiltrating leukocytes and the high expression of Anxa1 in the AqH and the neutrophils. After 48 h of EIU, the mast cells were intact, indicating granule re-synthesis, and there was a reduction of neutrophil transmigration and an increase in the number of mononuclear phagocytic cells in ocular tissues. Anxa1 expression was decreased in neutrophils but increased in mononuclear phagocytic cells. In the animals pretreated with c48/80 and subjected to EIU, mast cells responded to this secretagogue by degranulating and few transmigrated neutrophils were observed.

**Conclustions:**

We report that mast cells are a potential source of pharmacological mediators that are strongly linked to the pathophysiology of EIU, and the endogenous protein Anxa1 is a mediator in the homeostasis of the inflammatory process with anti-migratory effects on leukocytes, which supports further studies of this protein as an innovative therapy for uveitis.

## Introduction

Uveitis is a harmful inflammatory ocular condition that can be caused by infections and autoimmune diseases [1-3]. Although inflammation resolves in a few days, the recurrent nature of uveitis may result in other sight-threatening conditions such, as cataracts, glaucoma and even blindness [4,5].

Endotoxin-induced uveitis (EIU) is a widely accepted animal model for the study of acute ocular inflammation [6-8]. EIU is generally considered to be an inflammation of the anterior uvea, with some changes in the posterior segment (vitreous and retina) [9,10]. Exposure to exogenous bacterial toxins, such as lipopolyssacaride (LPS), stimulates ocular-resident cells to produce inflammatory cytokines, such as tumor necrosis factor (TNF)-α and other cytokines and inflammatory mediators [7,11].

Increased expression of inflammatory mediators leads to an infiltration of leukocytes that amplifies the inflammatory reaction in the eye [7,12]. Many of these inflammatory mediators are released by mast cells [13], which are highly specialized secretory cells, that play roles in both the innate and adaptive immune responses [14,15].

The major pathway for the activation of mast cells is the signaling cascade caused by the aggregation of high affinity receptors (FceRI) for immunoglobulin E (IgE), due to the binding of specific antigens [15]. An alternative way of activating mast cells is through exposure to a large number of polycationic molecules collectively known as the basic secretagogues of mast cells, such as synthetic compound 48/80 (c48/80) [16,17]. These agonists act as receptor mimetic agents that trigger mast cell degranulation by directly activating pertussis toxin (Ptx)-sensitive guanosine triphosphate inhibitory binding (Gi) proteins that control exocytosis [18-20].

Due to the fact that mast cells are essential for the resolution of bacterial infections through neutrophil mobilization to the site of infection [14], it has been suggested that their degranulation may contribute to the inflammatory process of EIU [21]. One of the endogenous mediators present in these mast cell granules is the protein Annexin A1 (Anxa1) [22].

Anxa1 is a member of a superfamily of annexin proteins that bind acidic phospholipids with high affinity in the presence of Ca^2+^ [23]. The interest in this protein has dramatically increased due to its potent anti-inflammatory and immunomodulatory properties [24]. In this regard, this endogenous mediator regulates acute [25] and chronic [22] inflammation. Anxa1 is a ubiquitous protein, and many inflammatory cells, in addition to mast cells, express this mediator, such as neutrophils [26], eosinophils [27], monocytes [28], and T cells [29].

This study indicates that mast cells are a potential source of pharmacological mediators that are, strongly linked to the pathophysiology of EIU. Additionally, this investigation indicates that the endogenous protein Anxa1 is a mediator in the homeostasis of the inflammatory process because it exhibits anti-migratory effects on leukocytes. The results indicate the requirement for further studies of this protein as an innovative therapy for uveitis.

## Methods

### Animals and endotoxin induced uveitis (EIU)

Male Wistar rats (São José do Rio Preto Medical School, São Paulo, Brazil) weighing 150-200 g (6 weeks old) were randomly distributed into 5 groups (n=10). The animals were housed in a 12 h light-dark cycle and were allowed food and water ad libitum. EIU was induced by a single subcutaneous injection of 0.2 mg *Escherichia coli* LPS endotoxin (Sigma Chemical Co. Poole, Dorset, UK) in 0.1 ml PBS into one hind footpad and the rats were euthanized at 24 h or 48 h after LPS injection. Control rats were injected with 0.1 ml PBS into the hind footpad.

### Compound 48/80 (c48/80) treatment

To further analyze the role played by mast cells in EIU, two groups of rats were treated with increasing doses of c48/80 (from 0.2 mg to 1 mg in 0.5 ml of PBS). The compound was administered twice daily, intraperitoneally, over 5 days. Then, one group of rats was treated to induce EIU and both groups of rats were euthanized after 24 h. All of the experiments were conducted in compliance with the ARVO Statement for the Use of Animal in Ophthalmic and Vision Research. The study was approved by the Ethics Committee in Animal Experimentation of the Federal University of São Paulo (São Paulo, Brazil; No. 0061/06).

### Histopathological evaluation

The right eyes of the rats were enucleated and fixed in a PBS solution containing 4% paraformaldehyde and 0.5% glutaraldehyde for 24 h at 4 °C, dehydrated by graded methanol and embedded in LRGold resin (London Resin Co., Reading, Berkshire, UK). Tissue sections (1 µm) were stained with toluidine blue. Infiltrating inflammatory cells in the anterior and posterior segments were evaluated by visual inspection in a blind fashion and counted with a high-power objective (40×) on an Axioskop 2-Mot Plus Zeiss microscope (Carl Zeiss, Jena, Germany). The mast cells, neutrophils and mononuclear phagocytes were distinguished by their specific morphological characteristics, including metachromatic cytoplasmic granules of mast cells and the nuclei of the leukocytes. The number of infiltrating inflammatory cells in 10 sections per eye, from five eyes from different animals, was averaged and recorded. Values were reported as the mean±SEM of number of cells-per-mm^2^.

### Infiltrating cells and total proteins concentration in AqH

Aqueous humor (AqH) was collected from the left eyes by anterior chamber puncture using a 29-gauge needle. The AqH pool was centrifuged and protein concentration in the supernatant was measured using a Bradford assay (Biorad, Hemel Hempsted, UK). For cell counting, the pellet was resuspended in PBS, and aliquots (100 µl) were diluted in Turk stain solution (1:10). The cells were counted with a Neubauer chamber (Laboroptik GmbH, Friedrichsdorf, Hessen, Germany) under a light microscope.

### Immunohistochemical studies

To detect Anxa1 protein expression, the eye sections (1 μm thick) were incubated with the following reagents at room temperature: Tris buffer 0.1 M, pH 5.4 (TBS) for 30 min; 3% hydrogen peroxide for 30 min and; 0.1% Tween-20 (Sigma-Aldrich Poole, Dorset, UK) diluted in TBS for 15 min. Non-specific binding sites were blocked with 10% BSA diluted in TBS (20 mM Tris buffer in 0.9% NaCl, pH 8.2) for 30 min. Sections were incubated overnight with a primary polyclonal rabbit anti-Anxa1 antibody (1:200; Zymed Laboratories, Cambridge, UK). After repeated washings in 1% PBS, a goat anti-rabbit IgG (Fc fragment-specific) antibody conjugated to 5 nm colloidal gold (1:50; British BioCell International, Cardiff, UK) was added. Silver enhancing solution (British BioCell International) was used to augment gold particle staining followed by hematoxylin counterstaining. Analysis was conducted with a Axioskop 2-Mot Plus Zeiss microscope (Carl Zeiss), using the AxioVision software (Zeiss).

### Western blotting analysis

AqH was sonicated in Tris-HCl solution (50 mM) containing phenylmethylsulphonilfluoride (1 mM, pH 7.4). The protein levels were determined by Bradford assay (Biorad, Hemel Hempsted, UK) and equalized before boiling in Laemmli buffer (4% SDS, 20% glycerol, 1 mM DTT, 2 mM EDTA and 1 mg/ml Coomassie brilliant blue). Equal amounts of protein (25 μg) were loaded onto a 10% sodium dodecyl sulfate-polyacrylamide gel for electrophoresis together with the appropriate molecular weight markers (Amersham Pharmacia Biotech, Buckinghamshire, UK) and transferred onto a Hybond-C extra nitrocellulose membrane (Amersham, Little Chalfont, UK). Membranes were blocked for 1 h with 5% non-fat dry milk diluted in TBS with 0.1% Tween-20 (TBST). Anxa1 expression was detected by an overnight incubation with the rabbit polyclonal Anxa1 antibody (1:1,000; Zymed). Equal loading was confirmed with an anti-α-tubulin antibody (1:2,000; Santa Cruz Biotechonology, Santa Cruz, CA). In both cases, the signal was amplified with peroxidase-conjugated goat anti-rabbit IgG (1:2,000; Serotec, Oxford, UK) diluted in TBST and visualized with an enhanced chemiluminescence kit (Amersham, Little Chalfont, UK). Then, densitometry was performed by the image analysis system (AxioVision Software; Zeiss).

### Statistical analysis

All data are the mean±SEM of n≥5 rats per group. Statistical differences between the means were determined by analysis of variance (ANOVA) followed, and if significant, by the Bonferroni post-hoc test. A probability value of less than 0.05 was considered significant.

## Results

### Analysis of inflammatory cells in ocular tissues in EIU

**Figure 1:**
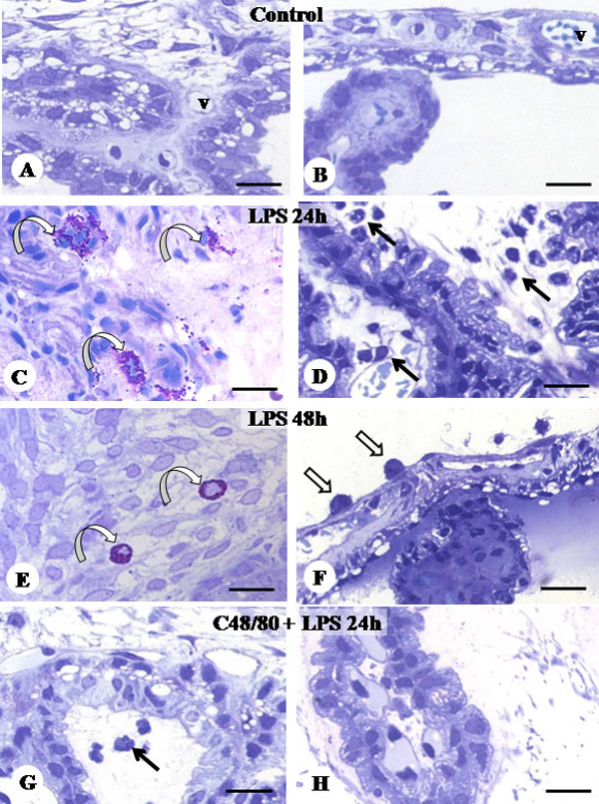
Histopathological analysis of the anterior eye segment. **A-H**: Light micrographs of rat eyes stained with toluidine blue. **A**, **B**: Ciliary body of control animals. **C**: Degranulated mast cell (curved arrow) in the ciliary body and extensive inflammatory cell infiltration in AqH (**D**), mainly neutrophils (arrows) at 24 h after endotoxin injection (LPS 24h). **E**: Intact mast cell (curved arrow) in the ciliary process and mononuclear phagocytic cells (open arrows) adhered to the iris surface (**F**) at 48 h post-injection (LPS 48h). **G**, **H**: Ciliary bodies of animals pretreated with secretagogue c48/80 before EIU (c48/80 + LPS 24h). Absence of mast cells and very few transmigrated inflammatory cells (arrow). V: blood vessel. Bars, 10 μm.

Initially, typical mast cells with intact granules were identified after staining with toluidine blue. In the control group, intact mast cells were observed mainly in the posterior eye segment (Table 1). Analysis 24 h after endotoxin injection showed a significant increase in the number of mast cells compared to the control group. These cells were observed in the process of degranulation in the ciliary body (100%; Figure 1C) and choroid (53%). In the later phase of inflammation at 48 h post-injection, a decrease of approximately 50% of the total number of mast cells was observed when compared to the group assessed after 24 h. All of the mast cells observed in the anterior (Figure 1E) and posterior eye segments were found to be intact. Mast cells were completely absent in the ocular tissues of all animals that received pretreatment with c48/80 (Figure 1G). The quantitative analyses of the mast cells in the eye are reported in Table 1.

**Table 1:** Quantitative analysis of mast cells in the eye.

**Groups**	**Anterior segment**		**Posterior segment**	
** **	**Mast cells**			
** **	**Intact**	**Degranulated**	**Intact**	**Degranulated**
Control	0.0	0.0	4.0±2	0.0
LPS 24h	0.0	15±3***	7.0±1.5	8.0±1.0
LPS 48h	12±3†††	0.0	5.0±2.0	0.0
C48/80	0.0	0.0	0.0	0.0
C48/80 + LPS 24h	0.0	0.0	0.0	0.0

Data are the mean±SEM of mast cells per mm^2^ analyzed in the ocular tissues of control animals, animals with EIU at the initial (LPS 24h) and late phases (LPS 48h), animals pretreated with c48/80 and animals pretreated with c48/80 and then subjected to endotoxemia (c48/80 + LPS24h). ***p<0.001 versus control, †††p<0.001 versus LPS 24h.

Histopathological and quantitative analyses revealed a large increase in the transmigration of inflammatory cells into the eye, mainly into the anterior segment, when assessed 24 h after LPS injection (Figure 1D; Table 2), whereas no infiltrating cells were observed in the eyes of control animals (Figure 1A,B; Table 2). Although the inflammatory process induced by LPS was most evident in the anterior eye segment, we observed changes in the posterior segment, including a significant increase of neutrophils in the choroid (p<0.001) and the retina (p<0.01; Table 3). In the late inflammatory response at 48 h post-injection, the number of neutrophils decreased significantly in the iris, ciliary body and choroid-retina complex (p<0.001; Table 2 and Table 3). However, a significant increase of mononuclear phagocytic cells was observed, and these cells were mainly adhered to the iris (Figure 1F; Table 2 and Table 3).

**Table 2:** Quantitative analysis of neutrophils and mononuclear phagocytic cells in the anterior eye segment.

**Groups**	**Iris**		**Ciliary body**	
** **	**Nϕ**	**Mϕ**	**Nϕ**	**Mϕ**
Control	0.0	0.0	0.0	0.0
LPS 24h	150±10***	25±5.0***	182±23***	0.0
LPS 48h	12±3.0†††	154±12†††	17±3.0†††	91±13†††
C48/80	0.0	0.0	0.0	0.0
C48/80 + LPS 24h	15±3.0‡‡	20±2.0‡‡	13±2.2‡‡	16±1.5‡‡‡

Data are the mean±SEM of neutrophils (Nϕ) and mononuclear phagocytes (Mϕ) per mm^2^ analyzed in the ocular tissues of control animals, animals with EIU at the initial (LPS 24h) and late phases (LPS 48h), animals pretreated with c48/80 and animals pretreated with c48/80 and then subjected to endotoxemia (c48/80 + LPS24h). ***p<0.001 versus control, †††p<0.001 versus LPS 24h; ‡‡p<0.01, or ‡‡‡p<0.001 versus C48/80.

**Table 3:** Quantitative analysis of neutrophils and mononuclear phagocytic cells in the posterior eye segment.

**Groups**	**Choroid**		**Retina**	
** **	**Nϕ**	**Mϕ**	**Nϕ**	**Mϕ**
Control	0.0	0.0	0.0	0.0
LPS 24h	85±7.0***	0.0	45±3.0**	0.0
LPS 48h	15±3.0†††	7.2±2.0†	32±4.0†	3.7±2.2
C48/80	0.0	0.0	0.0	0.0
C48/80 + LPS 24h	12±3.0‡‡	0.0	14±2.5‡‡	0.0

Data are the mean±SEM of neutrophils (Nϕ) and mononuclear phagocytes (Mϕ) per mm^2^ analyzed in the ocular tissues of control animals, animals with EIU at the initial (LPS 24h) and late phases (LPS 48h), pretreated with c48/80, and animals pretreated with c48/80 and then subjected to endotoxemia (c48/80 + LPS24h). **p<0.01 or ***p<0.001 versus control, †p<0.05 or †††p<0.001 versus LPS 24h; ‡‡p<0.01 versus C48/80.

The number of transmigrated leukocytes in the anterior and posterior segments at 24 h was significantly lower (p<0.001) in EIU animals pretreated with c48/80 and given LPS injection, compared to animals that had induced uveitis without pre-treatment (Figure 1H; Table 2 and Table 3). In the ocular tissues of animals only pretreated with the secretagogue, there were no inflammatory cells (Table 2 and Table 3).

### Analysis of the AqH in EIU

#### Total cell count

**Figure 2:**
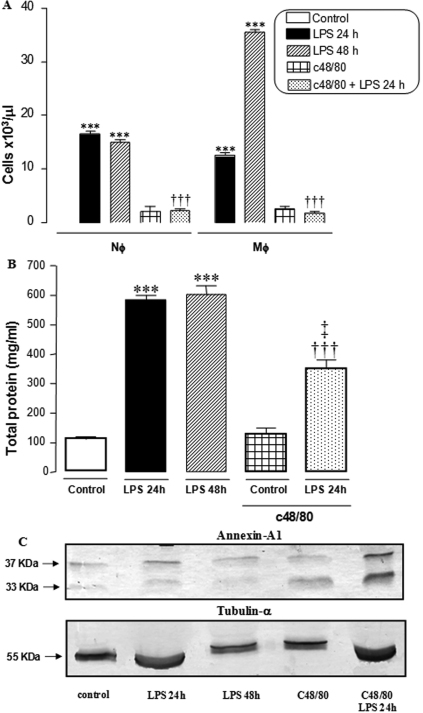
Cellular and protein extravasations into the AqH during EIU. **A**: Quantitative analysis of neutrophils (Nϕ) and mononuclear phagocytic cells (Mϕ) in the AqH. The results are expressed as number of cells per 10^3^/μl. ***p<0.001 versus control; †††p<0.001 versus LPS 24 h. **B**: The total protein concentration in the AqH are demonstrated in mg of protein/ml. ***p<0.001 versus control, †††p<0.001 versus LPS 24 h, ‡‡p<0.01 versus c48/80. **C**: Annexin A1 protein content was analyzed by western blotting and detected by the primary antibody rabbit anti-Anxa1. Two bands of 37 kDa and 33 kDa molecular weights were seen corresponding, to intact protein and NH_2_-terminal cleaved protein, respectively. The 55 kDa molecular weight tubulin-α was used as control. Control animals, animals at the initial (LPS 24 h) and late phase (LPS 48 h) of the inflammatory process, animals pretreated c48/80 (c48/80) only, or pretreated with c48/80 before EIU (c48/80 + LPS 24h). n=10 animals/group.

An influx of neutrophils (18±2.0×10^3^ cells/μl) and mononuclear phagocytic cells (14±1.5×10^3^ cells/μl) was observed in the AqH of the animals at 24 h after EIU. In the late inflammatory process, there was a reduction in the number of neutrophils (15±0.5×10^3^ cells/μl), whereas mononuclear phagocytic cells increased (35±2.2×10^3^ cells/μl) when compared to the initial phase. However, leucopenia was observed in the AqH of animals pretreated with c48/80, with or without the induction of uveitis (5±0.5×10^3^ cells/μl; Figure 2A).

#### Total proteins

Total proteins levels were evaluated at 24 and 48 h after endotoxin injection. At both 24 and 48 h, groups with EIU exhibited significantly higher protein levels (590±10 and 600±20 mg/ml, respectively) when compared with the control group (100±10). In the groups pretreated with c48/80, the protein levels were lower than in the initial inflammatory process at 24 h (350±20; Figure 2B).

### Anti-inflammatory protein Annexin-A1

Western blotting analysis of the AqH detected Anxa1 in all experimental groups. However, in animals pretreated with c48/80 and evaluated 24 h after uveitis induction, an increased immunoreactivity for Anxa1 was detected when compared with other experimental groups (Figure 2C).

### Annexin-A1 expression in inflammatory cells in EIU

**Figure 3:**
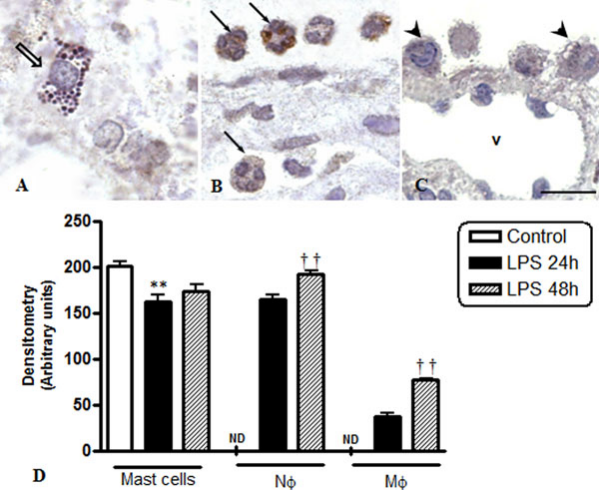
Anxa1 protein expression in inflammatory cells in EIU. Immunohistochemistry was performed to visualize the protein in (**A**) mast cells (open arrow) and (**B**) neutrophils (arrows) in the ciliary body at 24 h after EIU and (**C**) mononuclear phagocytic cells (arrowheads) adhered to the iris surface at 48 h after EIU. V: blood vessel. Counterstained with hematoxylin. Bars, 10 μm. **D**: Semiquantitative Anxa1 densitometric analysis. Data are the mean±SEM of the densitometric index (n=5 animals/group). *p<0.05 versus control group; †p<0.05 versus LPS 24 h. Neutrophils (Nϕ) and mononuclear phagocytic cells (Mϕ). ND, not detected.

Next we analyzed Anxa1 expression with a polyclonal anti-Anxa1 antibody which detects the intact (37 kDa) and cleaved (33 kDa) forms of the protein. Mast cells were immunoreactive for Anxa1 in ocular tissues from the control group, and in the tissues collected 24 (Figure 3A) and 48 h after endotoxin injection. Statistical analysis showed a significant reduction in Anxa1 expression in mast cells from the animals with EIU at 24 h (150±12) compared to control animals (200±10; Figure. 3D). Furthermore, no significant alteration in the Anxa1 expression in the late phase of inflammation (165±11) was observed when compared to the initial phase (Figure 3D).

In tests performed on neutrophils and mononuclear phagocytic cells, we observed immunoreactivity for Anxa1 at 24 and 48 h post-injection in the anterior (Figure 3B,C) and posterior eye segments. The statistical analysis showed significantly higher Anxa1 expression in neutrophils and mononuclear phagocytes at 48 h than at 24 h after uveitis induction (Figure 3D).

## Discussion

Experimental uveitis is widely used as a pre-clinical model for predicting drug efficacy in human settings. The involvement of mast cells has been evaluated in important eye diseases, such as conjunctivitis, atopic keratoconjunctivitis, and uveitis [30-32]. However, the role of mast cells in EIU has not been well studied. Thus, initially we investigated mast cells in rodent eyes to verify the presence, distribution, characteristics and participation of these cells in the intraocular inflammation after endotoxemia. We observed the recruitment and activation of mast cells in the experimental animals 24 h after the injection of the inflammatory stimulus of LPS, which coincided with the influx of leukocytes that occurred after the breakdown of the blood-ocular barrier, particularly in the anterior eye segment.

Mast cells secrete multiple mediators, including histamine, heparin, serine proteases, lipid-derived mediators and cytokines, which cause the clinical signs of inflammation [13,33]. When mast cells are challenged by an external stimulus, they respond by degranulation and the release vasoactive mediators [14,20] indicating that these cells influence the pathophysiology in experimental uveitis. The synthesis and release of eicosanoids and cytokines by mast cells are required for leukocyte transmigration into the site of inflammation [20,34-36]. In the late phase of the inflammatory response, 48 h after LPS administration, most mast cells present were found to be intact suggesting that these cells, after the initial activation, can re-synthesize the contents of their cytoplasmatic granules. Granular content re-synthesis after the secretion and extrusion of the cytoplasmatic granules was also studied in mast cells isolated from human lungs [37]. Our detailed histological analyses revealed a significant reduction of neutrophils, in contrast to the increase of the mononuclear phagocytic cells in ocular tissues, which was also seen in other studies [38].

The next step of our study was to evaluate mast cells in the process of leukocyte chemotaxis during ocular inflammation. For this purpose, we depleted mast cells in the ocular tissues by pre-treatment with the secretagogue c48/80. This compound acts on the cell surface, directly stimulating the activity of the GTPase associated with the membrane, which results in an increase in intracellular calcium [20,21,39] and in the depletion of intragranular mediators. Our results demonstrated that ocular tissue mast cells responded to c48/80 by degranulation and correspond to the connective tissue mast cells (CTMCs). The absence of mast cells, and thus the lack of the chemotactic mediators during EIU in ocular tissues, resulted in a reduction in the number of transmigrated leukocytes, confirming the importance of these mediators in chemotaxic process of inflammatory cells [35,36]. Furthermore, studies have shown that mast cell depletion by c48/80 resulted in a reduced number of neutrophils transmigrated into the peritoneal cavity of rats subjected to peritonitis [40].

Next, we analyzed the leukocytes and total protein in the anterior eye chamber after EIU. These analyses were conducted on the AqH and indicated a high increase in the transmigration of inflammatory cells and the leakage of plasma proteins at 24 and 48 h after EIU. In the literature, it has been demonstrated that the increased vascular permeability to plasma proteins is one of the main results of mast cell activation [41]. It is believed that, during uveitis histamine from degranulated mast cells plays a role in vasodilatation, increased vascular permeability, interstitial edema and in the expression of P-selectin on the membrane of endothelial cells, which then contributes to the recruitment of leukocytes [42]. Following with this hypothesis, a significant reduction occurred in the recruitment and migration of leukocytes and in the levels of plasma proteins in both AqH and ocular tissues in animals pre-treated with c48/80 before the induction of uveitis. For the same reasons, we wish to propose that the absence of pharmacological mediators from mast cells decreased vascular permeability, reducing leukocyte chemotaxis and proteins in the AqH. Previous studies performed in our laboratory showed that the preferential location of mast cells is in the anterior eye segment of birds, which indicates that the pharmacological mediators released from their cytoplasmatic granules may act physiologically in the AqH flow [43]. Moreover, our results suggest that after c48/80 treatment there was an increase in the level of Anxa1 in the AqH, and these data provide indirect confirmation that pro-and anti-inflammatory mediators are released from mast cells into the aqueous humor.

Because several lines of experimental evidence indicate the anti-inflammatory role of this protein [24] we examined Anxa1 expression in inflammatory cells (mast cells, neutrophils, and mononuclear phagocytes) during EIU. Anxa1 expression was different in the control and inflamed ocular tissues. At 24 h after LPS administration, a time period at which mast cells already showed signs of degranulation, mast cells expressed low amounts of Anxa1. Lower levels of Anxa1 were also found in nasal poliposis degranulated mast cells [44]. However, the first report of the presence of endogenous Anxa1 in mast cells [45] showed an increased concentration of the protein in the CTMCs of rat mesenteries after experimental inflammation and demonstrated its regulation after administration of the glucocorticoid dexamethasone. These findings indicate that these cells are a potential source of Anxa1 during the inflammatory process. In this regard, our results suggest that the release of mediators from mast cells during endotoxemia, including Anxa1, could act to inhibit the synthesis of pro-inflammatory mediators such as phospholipase A2, prostaglandin D2, and leukotriene E2 and stimulates the production of factors that contribute to the resolution of inflammation [24,46,47].

Finally, Anxa1 expression was also evaluated in leukocytes during inflammation. These investigations showed that neutrophils and mononuclear phagocytic cells expressed endogenous Anxa1 at 24 h, with a significant increase after 48 h. Our results indicated that the increase of endogenous Anxa1 expression in cells that had transmigrated to the inflammatory sites reduced the activation of these leukocytes, contributing to the resolution of inflammation, as shown in other studies [24,48]. Furthermore, the immunohistochemical and in situ hybridization studies demonstrated that the endogenous Anxa1 expression increased in the plasma membrane of circulating neutrophils during the inflammatory process and also in the cell cytoplasm of those cells after the transmigration [26]. These data indicated that Anxa1was regulated during leukocyte transmigration. In addition, other investigations have shown that the inflammatory process induced by zymozan exacerbated leukocyte transmigration into the peritoneal cavity in the absence of the Anxa1 protein, in Anxa1 knockout animals [49].

Regarding the role of Anxa1 in phagocytic mononuclear cells, the absence of the endogenous protein inhibited phagocytosis in 40% of the apoptotic polymorphonuclear cells [50]. Several studies have indicated the involvement of Anxa1 in the regulation of apoptosis in inflammatory cells, because its overexpression in monocytes increased apoptosis [51,52]. In addition, this protein has been implicated as a marker of cells undergoing apoptosis, possibly acting as an endogenous ligand for phosphatidylserine, facilitating the recognition, ingestion and, consequently, the removal of these cells by macrophages [53,54]. Based on these investigations and our observations of the increased expression of Anxa1 in neutrophils and phagocytic mononuclear cells during the late phase of inflammation, it is tempting to indicate that Anxa1 could also contribute to the resolution of the inflammatory process. However, further investigations are required for a better understanding of this issue.

### Conclusions

Based on the experimental model of uveitis, our results indicated that mast cells are a potential source of pharmacological mediators, that are strongly related to the pathophysiology of EIU, and that the endogenous protein Anxa1 is a key mediator in the homeostasis of the inflammatory process, showing regulatory action in leukocyte diapedesis. Thus, Anxa1 may be an innovative form of therapy for uveitis, by preventing the activation of mast cells and/or the infiltration of leukocytes in ocular tissues.
